# The COVID Connection: Pandemic Anxiety, COVID-19 Comprehension, and Digital Confidence

**DOI:** 10.1177/00027642211003155

**Published:** 2021-11

**Authors:** Laura Robinson, Jeremy Schulz, Øyvind N. Wiborg, Elisha Johnston

**Affiliations:** 1 Santa Clara University, Santa Clara, CA, USA; 2 University of California Berkeley, Berkeley, CA, USA; 3 University of Oslo and Oslo Metropolitan University, Oslo, Norway; 4 El Camino College, Torrance, CA, USA

**Keywords:** COVID-19, digital divide, anxiety, confidence, vulnerability

## Abstract

This article presents logistic models examining how pandemic anxiety and COVID-19 comprehension vary with digital confidence among adults in the United States during the first wave of the pandemic. As we demonstrate statistically with a nationally representative data set, the digitally confident have lower probability of experiencing physical manifestations of pandemic anxiety and higher probability of adequately comprehending critical information on COVID-19. The effects of digital confidence on both pandemic anxiety and COVID-19 comprehension persist, even after a broad range of potentially confounding factors are taken into account, including sociodemographic factors such as age, gender, race/ethnicity, metropolitan status, and partner status. They also remain discernable after the introduction of general anxiety, as well as income and education. These results offer evidence that the digitally disadvantaged experience greater vulnerability to the secondary effects of the pandemic in the form of increased somatized stress and decreased COVID-19 comprehension. Going forward, future research and policy must make an effort to address digital confidence and digital inequality writ large as crucial factors mediating individuals’ responses to the pandemic and future crises.

## Overview

In this research we explore the ever-expanding frontier of digital inequality by probing the role of digital confidence as a contributor to two outcomes: comprehension of information about COVID-19 and anxiety related to the virus as manifested in physical symptoms. Probing these connections with a nationally representative data set of American adults, we find that digital confidence predicts these experiential outcomes, even when models factor in sociodemographic, psychological, and socioeconomic vulnerabilities. As the findings show, individuals lacking digital confidence are less likely to feel that they comprehend COVID-19 information *and* are more likely to manifest physical symptoms of anxiety (sweating, trouble breathing, nausea, or a pounding heart) when merely thinking about their experiences with the COVID-19 outbreak. These results are the first to establish a linkage between digital inequality and the presence of somatized anxiety related to COVID-19. The findings thereby provide a foundation for tying digital inequalities to crisis comprehension and forms of distress directly implicated in bodily well-being.

## Building the Bridge: The COVID-19 Pandemic and Digital Inequality

Only a year old, the COVID-19 pandemic has already served as the focus of several research projects geared toward uncovering its effects on both anxiety and comprehension. For example, recent research has already yielded evidence that the pandemic has triggered and exacerbated anxiety, depression, and fear (Holmes et al., 2020). Several recent original surveys administered during the early stages of the pandemic have found that respondents’ levels of distress vary along with both social and psychological vulnerabilities in different national settings (Fitzpatrick et al., 2020; Petzold et al., 2020). Research focused on the consequences of the crisis for anxiety and comprehension, however, has neglected to consider the role of digital inequalities in mediating these secondary effects of the pandemic. Studies of anxiety, for instance, have restricted their explanatory variables to indicators of social and economic vulnerabilities (such as age, race/ethnicity, income, gender, and partner status) and general psychological disposition. This neglect of digital inequalities is evident, even though the COVID-19 pandemic can be considered the very first truly global health event taking place since the inception of the information age (Castells, 1996-2000).

However, there is good reason for exploring the role of digital inequality as a key factor channeling the effects of the pandemic. As COVID-19 continues to prompt lockdowns, curfews, and stay-at-home orders, digital resources have become even more critical as the primary lifeline for those with access to the internet for telework, eLearning, telehealth, eCommerce, and so on. During the pandemic, the lack of home broadband and/or smartphone dependence can be particularly problematic. Programs such as Zoom are the new *sine qua non* for eLearning, telework, and a host of essential activities. Those who lack home broadband or who are smartphone dependent may find themselves making do with inadequate stopgap measures. Parents and workers often must drive to parking lots where they can pick up signals from public WiFi networks, winding up working or learning in their car in areas far from their homes. In April of 2020, 40% of lower income families in the United States were forced to rely on public Wi-Fi for schoolwork due to lack of reliable home internet connection. In addition, 43% of children in lower income families in the United States had no choice but to use smartphones to complete their schoolwork (Vogels et al., 2020), potentially engendering learning gaps with lifelong consequences.

Over a year into a pandemic—which has made digital resources ever more indispensable for work, school, and consumption—many Americans still do not enjoy equal access to the internet and/or the skills to use digital resources effectively in a number of life realms. Even in highly connected and developed societies such as the United States, not all individuals have equal access to digital resources, the digital skills, or equal confidence deploying digital technologies. In May of 2020, the Pew Research Center reported that over 20 million Americans lacked broadband internet access of any speed or quality. Further, up to 162 million Americans are not using the internet at high broadband speeds (Microsoft, 2019). Simply put, up to half of the total population of the United States lacks consistently high-quality access to the digital resources; these resources are critical to sustaining well-being and life chances during the crisis engendered by the COVID-19 pandemic. For many, digital disadvantage represents a dire hardship with potentially devastating effects.

While the pandemic has brought the “digital divide” into our national conversation to a greater extent than ever before, it has long been studied by scholars of digital inequality. Researchers have identified three main levels of digital inequality (van Dijk, 2005; Witte & Mannon, 2010), all of which are particularly consequential during the COVID-19 pandemic. While first-level digital inequalities encompass resource inequalities in terms of hardware or network access, second-level digital inequalities concern digital skills; third-level digital inequalities are responsible for disparities in offline/behaviors and conditions (Gui & Büchi, 2019; Helsper, 2012; Ragnedda, 2017; Robinson et al., 2018; Robinson et al., 2020c). All levels of the digital divide have been tied to sociodemographic disparities including age, gender, race/ethnicity, income, and education (Pew Research Center, 2019).

Studies are just beginning to put existing work on digital inequalities into dialogue with research on the pandemic, not only in terms of COVID-19’s primary effects on exposure risk and health (Robinson et al., 2020d), but also in terms of its secondary impacts on individuals’ distress. An early exploratory study put forward the idea that digital inequalities may influence the individual-level impacts of the fallout from the COVID-19 crisis in terms of outcomes such as social isolation and anxiety (Robinson et al., 2020a). Another study raised the theoretical possibility that digital resources and activities may impact anxiety or mental health, insofar as individuals may react to the pandemic with high levels of emotional distress, fear, and confusion (Beaunoyer et al., 2020). Other research has shown how digital inequalities make access to vital services such as health care via telemedicine (Khilnani et al., 2020), telework and eLearning (Robinson et al., 2020b), and digital communications (Nguyen et al., 2020) less accessible for already vulnerable segments of the population during the pandemic. Therefore, given the strong possibility of digital inequality contributing to diminished well-being engendered by the pandemic, we take on the task of analyzing the pandemic’s secondary outcomes in relation to digital inequalities with nationally representative data from the United States.

## Research Questions and Hypotheses

Given the potential connections between ongoing digital inequalities and the COVID-19 pandemic, the links between digital inequalities and the effects of the pandemic warrant empirical investigation. We therefore pose two questions amenable to empirical analysis, namely whether and to what extent digital inequalities affect (1) pandemic anxiety stemming from individuals’ experiences and (2) information comprehension about COVID-19. To more fully understand the connections between these two outcomes and digital inequality, we make use of the concept of digital confidence operationalized by the Pew Research Center in its American Trends Panel (ATP) survey. Digital confidence is taken from the Pew Research Center’s measure of “digital savviness” that incorporates frequency of internet use, comfort using the internet, and confidence in one’s own digital abilities.

The first orienting research question takes up the challenge of identifying potential associations between digital confidence and pandemic anxiety. We ask the following: *Controlling for sociodemographic and general anxiety vulnerabilities, can digital inequalities predict anxiety induced by the pandemic among adults in the United States during the first wave of the COVID-19 pandemic?* To advance this inquiry, we test the following hypothesis:

**Hypothesis 1:** Hypothesis regarding pandemic-related anxiety.

Respondents who self-identify as digitally confident will exhibit lower probability of experiencing anxiety induced by the pandemic, relative to respondents who self-identify as digitally underconfident, net of control variables for sociodemographic vulnerabilities, general anxiety, and socioeconomic status.

In tandem, we also explore potential links between digital confidence and COVID-19 comprehension with the second orienting research question. We ask the following: *Controlling for sociodemographic and general anxiety vulnerabilities, can digital inequalities predict COVID-19 comprehension among adults in the United States during the first wave of the COVID-19 pandemic?* To advance this inquiry, we test the following hypothesis:

**Hypothesis 2:** Hypothesis regarding comprehension of COVID-19 crisis.

Respondents who self-identify as digitally confident will exhibit higher probability of reporting “having a handle” on the COVID-19 crisis, relative to respondents who self-identify as digitally underconfident, net of sociodemographic vulnerabilities, general anxiety, and socioeconomic status.

We assess both of these hypotheses through an examination of a nationally representative data set of adults in the United States collected in April of 2020 during the early stages of the crisis.

## Data Source: Wave 66 of the American Trends Panel

To test these hypotheses, we analyze data from Wave 66 of the Pew Research Center’s ATP survey, a nationally representative survey of adults in the United States in April of 2020 (Pew Research Center, 2019).^1^ The ATP survey is well-suited to answering our research questions as it includes questions capturing both pandemic anxiety and COVID-19 comprehension, as well as measurements of respondents’ digital confidence or “digital savviness.”

Wave 66 of the ATP survey included 10,139 individual respondents selected from an address-based directory of U.S. households. The cumulative response rate, as reported by the Pew Research Center, was an extremely high 92%. As we discuss below, we used the customized survey weights devised by Pew in order to account for the multistep sampling design and to yield estimates which accurately represented the U.S. adult population.

This wave of the ATP was administered via the internet with tablets provided by Pew to respondents who lacked their own digital equipment. The provision of tablets may have mitigated device divides or obviated smartphone dependence (Tsetsi & Rains, 2017) where the survey administration was concerned. However, where our study is concerned, it is important to note that Pew did not report providing any training geared toward helping respondents use these devices effectively. The administration of the ATP thus compensates to some extent for first-level device digital inequalities, but does not mitigate second-level digital skill inequalities to any extent.

## Outcome Variable: Pandemic Anxiety

We draw on several ATP survey items to operationalize the outcome variables. The first dependent variable, pandemic anxiety, is based on the survey item that captures the frequency of physical symptoms of anxiety connected with the COVID-19 outbreak. The relevant survey item asks how often the survey respondents experience physical symptoms of anxiety during a given week when reflecting on their experience with the COVID-19 outbreak phrased as follows: “In the past 7 days, how often have you had physical reactions, such as sweating, trouble breathing, nausea, or a pounding heart, when thinking about your experience with the coronavirus outbreak?” The four ordinal response categories offered by the survey are the following: “Rarely or none of the time (less than 1 day),” “Some or a little of the time (1-2 days),” “Occasionally or a moderate amount of time (3-4 days),” or “Most or all of the time (5-7 days).” This question was adapted from the “Impact of Event Scale” used by the American Psychiatric Association to capture reported physical distress caused by traumatic events. The criteria used are defined in the *Diagnostic and Statistical Manual of Mental Disorders–Fourth Edition* as symptoms of posttraumatic stress disorder. We dichotomize this categorical outcome variable to perform logistic regressions. The dichotomization proceeds by first retaining the lowest level of the variable, recoded as zero. Then the three highest levels of the variable are collapsed into a single level, coded as one. The dichotomized variable indicates whether a respondent experiences pandemic anxiety more frequently than rarely or none of the time. In the final section of the results, through ordinal logistic modeling we establish that consistent results are obtained when the outcome is retained in its raw form as an ordered four-category response variable.

## Outcome Variable: COVID-19 Comprehension

The second dependent variable is defined as COVID-19 comprehension. This variable derives from a single survey item asking respondents whether or not they comprehend information surrounding the virus outbreak. The question is phrased in the following form: “In general, do you feel like you have a handle on the issues and developments surrounding the coronavirus outbreak?” The two response categories offered by the survey are the following: “Yes, I feel like I have a handle on the issues and developments surrounding the coronavirus outbreak” and “No, I feel like I can’t get a handle on the issues and developments.” The variable is coded in a binary way in its raw form. Affirmative answers to this question are coded as one, while negative answers are coded as zero.

## Explanatory Variable: Digital Confidence

The explanatory variable is digital confidence as developed by the Pew Research Center by bringing together frequency of internet use and comfort using digital technologies. This measure has been employed to study political awareness and trust in the media (Kauth, 2020; Mitchell et al., 2018). In this research, we utilize the digital confidence measure to examine an outcome with similarities to political awareness, namely crisis comprehension associated with COVID-19. In addition, we extend its use to a new domain: pandemic anxiety.

As it is constructed by Pew, the variable digital confidence is an index measure constructed in a combinatory fashion out of two separate items: frequency of internet use and confidence in one’s digital abilities. We call this variable “digital confidence” in deference to Pew’s characterization of the survey item. Pew Research Center (2020) offers the following description of this measure:

The variable “Digital confidence” in the online tool is a measure of level of use and comfort with digital technologies. It is based on responses to two questions: (1) Reporting using the internet at least multiple times a day and (2) Being very confident in one’s ability to use electronic devices.

Based on the answers to both questions, Pew assigns respondents to one of the following three groups: The digitally “savvy” (the digitally confident), digital “dabblers” (the digitally intermediate), and the digitally “disengaged” (the digitally underconfident). The digitally confident satisfy both of Pew’s criteria: (1) they report “using the internet multiple times per day” and (2) they report a “high level of comfort/confidence in their own ability to use electronic devices.” The digitally intermediate are classified as respondents who satisfy one but not both of the criteria. The digitally intermediate respondents might use the internet multiple times per day but not express a high level of comfort/confidence or vice versa. Finally, the digitally underconfident are respondents who neither use the internet multiple times a day nor report a high level of comfort/confidence in their ability to use devices.

## Control Variables

The models incorporate three kinds of controls comprised of the following: (1) sociodemographic variables, (2) socioeconomic status variables, and (3) general anxiety. As potential confounders, these variables may be expected to exhibit correlations with digital confidence, the focal explanatory variable, as well as one or both of the outcomes. The sociodemographic control variables are common in studies of digital inequality and health/anxiety: age, gender, race/ethnicity, partner status, and metropolitan status (Ball et al., 2019; Cotten et al., 2012). In particular, these sociodemographic variables have been used as controls in models devised to predict mental health, depression, and anxiety related to the pandemic in the United States (Fitzpatrick et al., 2020; Nimrod, 2020). In addition, the socioeconomic status variables of income and education have been utilized extensively in digital inequality research (Blank & Reisdorf, 2012; Eastin & LaRose, 2000). Finally, general anxiety serves as a proxy for general psychological vulnerability (Barrett, 2000; Celik & Yesilyurt, 2012). The incorporation of general anxiety makes it possible to distinguish between long-term distress and those secondary effects specifically associated with the pandemic (Petzold et al., 2020; Sun et al., 2020).

The following sociodemographic controls are included in the models:

*Age*: Age is specified in the ATP as membership in one of the following three age groups: (1) 18-29 years (baseline category); (2) 30-49 years; (3) 50-64 years; and (4) 65 years or older.

*Gender*: Gender is specified in the ATP as either (1) Female (baseline category) or (2) Male.

*Race/ethnicity*: Race/ethnicity is specified in the ATP as membership in one of the following four categories: (1) White non-Hispanic (baseline category), (2) Black non-Hispanic, (3) Hispanic, or (4) other.

*Partner status*: Partner status is specified in the ATP as membership in one of the following categories: (1) Married, (2) Widowed, (3) Divorced, (4) Separated, (5) Living with a partner, or (6) Never been married. To facilitate model interpretation, we collapse the six original marital status categories into partnered (baseline category: includes married and living with a partner) and partnerless (widowed, divorced, separated, never been married).

*Metropolitan status*: Metropolitan status is specified in the ATP as either (1) metropolitan (baseline category) or (2) nonmetropolitan residence.

In addition to the sociodemographic controls, the following two socioeconomic status variables and general anxiety are also added to the models:

*Income level*: Income Level is specified in the ATP as membership in one of the following three annual income bands: (1) Earning less than $30,000 (baseline category), (2) Earning between $30,000 and $74,999, or (3) Earning $75,000 or more.

*Educational Achievement*: Educational achievement is specified in the ATP as membership in one of the following six groups: (1) Less than high school; (2) High school graduate; (3) Some college, no degree; (4) Associate degree (2-year degree); (5) College degree/some post-grad; and (6) Postgraduate. We treat this factor as an ordered categorical variable and recode it into the following three groups for ease of model interpretation: (1) High school degree or less (baseline category); (2) 2-year college (some college, no degree, associate degree; and (3) 4-year college degree or higher (includes college degree/some post-grad, postgraduate).

*General Anxiet*y: General Anxiety is specified in the ATP with the following question: “In the past 7 days, how often have you . . . felt nervous, anxious, or on edge?” The response categories are four frequency categories: (1) Rarely or none of the time (less than 1 day; baseline category); (2) Some or a little of the time (1-2 days); (3) Occasionally or a moderate amount of time (3-4 days); or (4) Most or all of the time (5-7 days).

## Analytic Data Set and Descriptive Statistics

To facilitate the modeling, we created a primary analytic data set composed of 9,404 participants. This analytic data set was generated through the listwise deletion of 735 (7.2%) respondents with at least one response of missing, refused, or NA on any of the variables in the raw data set. Table 1 reports the characteristics of the remaining respondents for each of the two outcome variables as well as all of the explanatory and control and variables.^2^

**Table 1.** Descriptive Statistics.

	Percentage	
	Unweighted	Weighted
*Digital confidence*		
Digitally underconfident (disengaged)	10	14
Digitally intermediate (dabblers)	31	30
Digitally confident (savvy)	59	56
*Pandemic anxiety*		
Rarely or none of the time (less than 1 day)	84	82
Some or a little of the time (1-2 days)	11	11
Occasionally or a moderate amount of time (3-4 days)	4	5
Most or all of the time (5-7 days)	1	1
*COVID-19 comprehension*		
No, I feel like I can’t get a handle on the issues and developments	12	15
Yes, I feel like I have a handle on the issues and developments	88	85
*Age, years*		
18-29	11	20
30-49	32	34
50-64	31	26
65+	27	20
*Gender*		
Male	45	49
Female	55	51
*Race/ethnicity*		
White non-Hispanic	69	65
Black non-Hispanic	8	11
Hispanic	17	16
Other	6	9
*Partner status*		
Partnerless	36	43
Living with a partner	64	57
*Metropolitan status*		
Nonmetropolitan	11	13
Metropolitan	89	87
*Income group*		
<$30,000	19	30
$30-$74,999	34	36
$75,000+	47	34
*Education*		
4 Years or college or more	56	32
2 Years of college	30	32
High school graduate or less	14	36
*General anxiety*		
Rarely or none of the time (less than 1 day)	34	34
Some or a little of the time (1-2 days)	33	32
Occasionally or a moderate amount of time (3-4 days)	22	22
Most or all of the time (5-7 days)	11	13
Number of participants with complete data: 9,404		

We conducted bivariate chi-square tests for associations between the two outcomes and the explanatory and control variables. With respect to the first outcome pandemic anxiety, we find that the following control variables exhibit a statistically significant bivariate association: age, gender, race/ethnicity, partner status, metropolitan status, income, education, and general anxiety. However, we find that the explanatory variable, digital confidence, does not demonstrate a significant association with pandemic anxiety in a bivariate chi-square test. As we discuss later, this important association is masked by age. This finding is in keeping with previous studies on age (Phillips, 1989).

Turning to the second outcome, COVID-19 comprehension has a statistically significant bivariate association with all of the controls used on the primary models, namely age, gender, race/ethnicity, partner status, metropolitan status, income, education, and general anxiety. With regards to COVID-19 comprehension, the explanatory variable, digital confidence, is significant (*p* = .05) in the bivariate context.

## Modeling and Specification Strategy: Multiple Survey-Weighted Logistic Regressions

The analytic strategy employed to build the primary models is designed to evaluate the effects of digital confidence as a contributor to two different outcomes: (1) physical symptoms of pandemic anxiety and (2) COVID-19 comprehension defined as individuals’ capacity to grasp or “handle” the COVID-19 crisis. To focus on the effects of digital confidence as an explanatory variable, we estimate a series of survey-weighted logistic regression models. Following Hosmer and Lemeshow (2000), we express the baseline logistic model as the following:

$$\pi \left(x\right)=E(Y\;|\;x)=\frac{e^{\beta_{0}+\beta_{1}x_{1}+...+\beta_{k}x_{k}}}{1+e^{\beta_{0}+\beta_{1}x_{1}+...+\beta_{k}x_{k}}}$$

In this equation, *Y* is treated as a binary random variable (values: 0, 1). We represent the intercept with *β_0_*. We represent each coefficient of the independent variables with *β_1_* through *β_k_*. Since all of the predictors are categorical, we represent each level using dummy variables.

To enhance model interpretability, we also compute average marginal effects (AME) (Allison, 1999; Mood, 2009).^3^ Interpretations of the AME correspond approximately to interpretations of marginal effects obtained by linear probability models. The AME can be understood as the predicted change in the probability of the outcome (Y), conditional on marginal changes in the predictor (X), averaged across all observations in the sample. For categorical predictors represented by a set of dummy variables, the AME of each dummy variable represents the difference in the predicted probability between individuals in the specified category and their counterparts in the reference category.

We follow the same specification strategy for the two outcome variables: pandemic anxiety and COVID-19 comprehension. For each outcome, we incrementally build four weighted logistic regression models. Each of the models feature particular packages of control variables outlined earlier in the paper. Model 1, the baseline model, is a weighted bivariate logistic model that estimates the relationship between the outcomes and the explanatory variable of digital confidence, without any controls. We then introduce sociodemographic controls (age, gender, race/ethnicity, partner status, and metropolitan status) in the next model, Model 2. In Model 3, we then add the variable general anxiety to take into account the general level of anxiety experienced by the respondents. For each of the two outcomes, the full model, Model 4, adds the two socioeconomic status variables to the model alongside the sociodemographic controls and general anxiety.

In all logistic models, we follow the guidelines provided by the Pew Research Center for implementing its customized survey design weights developed to account for the sampling design of the ATP. Therefore, we use an estimator relying on a quasi-likelihood approach to logistic regression that provides weighted linearized standard errors appropriate to Pew’s sampling strategy (Archer & Lemeshow, 2006). This approach provides unbiased estimates when used in conjunction with this complex survey sampling design. Since this estimator does not compute the standard chi-square statistics obtained by the maximum likelihood estimator (Hosmer & Lemeshow, 2000), we rely on a specially developed means-residual *F*-test to calculate goodness of fit statistics for each model (Archer & Lemeshow, 2006).

## Findings: Pandemic Anxiety

### Results: Digital Confidence Across Models

We now turn to the results relating to the explanatory variable digital confidence across the different model specifications. In Tables 2 and 3, we see that digital confidence has a statistically significant relationship with pandemic anxiety across all three models incorporating control variables. Though the odds ratio coefficients diverge slightly from the AME coefficients, the estimates are statistically significant across both types of coefficients. Across Models 2, 3, and 4, we can discern a statistically meaningful difference between members of the group with the highest digital confidence (the digitally confident) and members of the group with the least digital confidence (the digitally underconfident). This is the case whether the coefficients are calculated as odds ratios or as AMEs.

**Table 2.** Logistic Regression: Pandemic Anxiety Dependent on Digital Confidence (Odds Ratios).

	(1)	(2)	(3)	(4)
	Baseline	Sociodemographic	Sociodemographic + anxiety	Full model
Digitally Intermediate	0.843 (0.134)	0.705* (0.115)	0.704 (0.130)	0.766 (0.143)
Digitally Confident	0.840 (0.125)	0.577*** (0.093)	0.526*** (0.097)	0.601** (0.114)
Age, years				
30-49		0.849 (0.109)	0.894 (0.125)	0.936 (0.132)
50-64		0.523*** (0.073)	0.608*** (0.091)	0.629** (0.095)
65+		0.305*** (0.055)	0.416*** (0.080)	0.431*** (0.083)
Male		0.536*** (0.052)	0.683*** (0.071)	0.690*** (0.072)
Black non-Hispanic		1.035 (0.168)	1.281 (0.225)	1.206 (0.212)
Hispanic		1.056 (0.141)	1.172 (0.171)	1.068 (0.158)
Race/ethnicity: Other		1.102 (0.194)	1.144 (0.224)	1.157 (0.227)
Partnerless		1.200* (0.111)	1.057 (0.107)	0.995 (0.108)
Nonmetropolitan		0.702* (0.116)	0.645* (0.120)	0.601** (0.115)
Anxiety				
Most or all of the time (5-7 days)			17.869*** (3.535)	17.486*** (3.495)
Occasionally or a moderate amount of time (3-4 days)			8.496*** (1.595)	8.521*** (1.599)
Some or a little of the time (1–2 days)			3.291*** (0.631)	3.336*** (0.640)
Income				
$30-$74,999				0.864 (0.115)
$75,000+				0.767 (0.107)
Education				
2 Years college				0.813 (0.108)
4 Years college				0.802 (0.101)
Observations	9,404	9,404	9,404	9,404
*F*	0.713	11.701	27.699	21.733
*p*	.490	.000	.000	.000
Survey-F (Archer-Lemeshow Test)	0.000	1.505	1.015	0.459
Survey-P (Archer-Lemeshow Test)	1.000	0.140	0.425	0.902

*Note*. Exponentiated coefficients. Linearized standard errors derived from survey-weighted logistic regressions (in parentheses).

* *p* < .05. ^**^p < .01. ^***^p < .001.

**Table 3.** Logistic Regression: Pandemic Anxiety Dependent on Digital Confidence (Average Marginal Effects).

	(1)	(2)	(3)	(4)
	Baseline	Sociodemographic	Sociodemographic + Anxiety	Full model
Digitally intermediate	−0.026 (0.025)	−0.056* (0.027)	−0.048 (0.027)	−0.036 (0.026)
Digitally confident	−0.026 (0.023)	−0.083** (0.027)	−0.084** (0.026)	−0.065* (0.026)
Age, years				
30-49		−0.028 (0.022)	−0.015 (0.019)	−0.009 (0.019)
50-64		−0.097*** (0.022)	−0.063** (0.020)	−0.058** (0.020)
65+		−0.152*** (0.023)	−0.101*** (0.022)	−0.096*** (0.022)
Male		−0.086*** (0.013)	−0.046*** (0.012)	−0.045*** (0.012)
Black non-Hispanic		0.005 (0.023)	0.031 (0.023)	0.023 (0.023)
Hispanic		0.008 (0.019)	0.019 (0.018)	0.008 (0.018)
Race/ethnicity: Other		0.014 (0.026)	0.016 (0.025)	0.018 (0.025)
Partnerless		0.026 (0.013)	0.007 (0.012)	−0.001 (0.013)
Nonmetropolitan		−0.045* (0.019)	−0.049** (0.019)	−0.056** (0.019)
Anxiety				
Most or all of the time (5-7 days)			0.392*** (0.025)	0.384*** (0.025)
Occasionally or a moderate amount of time (3-4 days)			0.230*** (0.017)	0.230*** (0.017)
Some or a little of the time (1-2 days)			0.086*** (0.012)	0.088*** (0.012)
Income				
$30-$74,999				−0.018 (0.017)
$75,000+				−0.032 (0.017)
Education				
2 Years college				−0.026 (0.017)
4 Years college				−0.027 (0.016)
Observations	9,404	9,404	9,404	9,404
*F*	0.713	11.701	27.699	21.733
*p*	.490	.000	.000	.000
Survey-F (Archer–Lemeshow Test)	0.000	1.505	1.015	0.459
Survey-P (Archer–Lemeshow Test)	1.000	0.140	0.425	0.902

*Note*. Linearized standard errors derived from survey-weighted logistic regressions (in parentheses).

* *p* < .05. ^**^p < .01. ^***^p < .001.

Importantly, the model indicates that the sociodemographic variable age, which is highly correlated with the outcome of pandemic anxiety on its own, masks the effect of digital confidence in the baseline model (Model 1), which does not adjust for any other potentially confounding factors. Once age and other sociodemographic factors are taken into account, digital confidence emerges as significant across all models. In Model 2 (the sociodemographic model), the odds of the digitally confident experiencing pandemic anxiety are 0.577 times that of the digitally underconfident (*p* < .001). The specific masking effect of age in relation to digital confidence as an explanatory variable is also substantiated in an analysis separate from the primary models.

When general anxiety is added in Model 3, the effect of digital confidence is slightly weakened, but remains of roughly similar size (odds ratio [*OR*] = 0.526 AME = −0.084, *p* < .01). In the full model, when the socioeconomic variables are included in the model alongside sociodemographic controls and general anxiety, digital confidence continues to exhibit a statistically significant relationship with pandemic anxiety. Here, the digitally confident have lower odds (*OR* = 0.601, *p* < .01, Model 4) of experiencing pandemic anxiety than the digitally underconfident (while those in the intermediate category are statistically no different than members of the reference group). Expressed in terms of AMEs, the digitally confident have a 6.5% lower probability of reporting pandemic anxiety as contrasted with their digitally underconfident counterparts (AME = −0.065, *p* < .05, Model 4).

**Figure 1.**
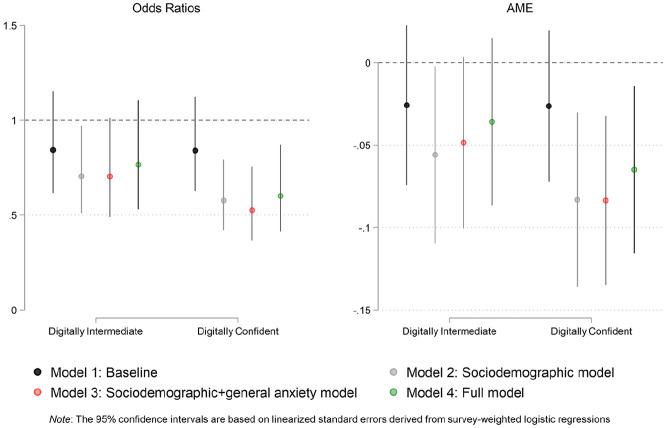
Coefficient plots for survey-weighted logistic models (pandemic anxiety).

Figure 1 represents the coefficients associated with both levels of the digital confidence variable across all four models in terms of both odds ratios (left panel) and AMEs (right panel). As the plot makes clear, the statistically meaningful gap is between members of the group with the highest digital confidence and members of the group with the least digital confidence.

### Results: Control Variables in Full Model

In Tables 2 and 3, we present the odds ratio estimates and AMEs derived from the four survey-weighted logistic regression models designed to predict the dichotomized outcome of pandemic anxiety as a function of the explanatory variable digital confidence and all control variables. Turning to the results interpreted in terms of odds ratios, we inspect the results from the full model. We see that race/ethnicity, partner status, income, and education are not statistically significant in the full model, whether coefficients are given in terms of odds ratios or AMEs.

We see that the following control variables exhibit statistically discernable associations with the outcome of pandemic anxiety: age, gender, metropolitan status, and general anxiety; all predict the odds of experiencing pandemic anxiety in the full model. More specifically with respect to age, in terms of odds ratios, relative to those in the baseline age bracket of 18 to 29 years, respondents in the 65+ years age bracket have .431 times the odds of reporting physical symptoms of pandemic anxiety (*p* < .001). The odds of male respondents belonging to the pandemic-anxious group are 0.690 (*p* > .001) times the odds of their female counterparts. Likewise, respondents who do not live in metropolitan areas have lower odds (*OR* = 0.601, *p* < .01, Model 4) than their metropolitan counterparts of experiencing pandemic anxiety. Finally, compared with the baseline group (those with a high school education or less), both groups of respondents with higher levels of education have lower odds of experiencing pandemic anxiety.

Perhaps unsurprisingly, the variable general anxiety has a strong statistically significant association with the outcome variable pandemic anxiety. Compared with the baseline group (those who are rarely or never anxious), members of all other categories of general anxiety frequency have higher odds of experiencing pandemic anxiety. This applies to those who experience some general anxiety (*OR* = 3.3, *p* < .001, Model 4), occasional general anxiety (*OR* = 8.5, *p* < .001, Model 4), and general anxiety most of the time (*OR* = 17.48, *p* < .001, Model 4).

## Findings: COVID-19 Comprehension

### Results for COVID-19 Comprehension: Digital Confidence Across Models

With respect to COVID-19 comprehension, digital confidence has a consistently statistically discernable effect on the outcome across all four models, including the baseline model. The association between the explanatory variable digital confidence and COVID-19 comprehension is consistently positive and statistically significant, whether coefficients are expressed as odds ratios or AMEs. More specifically, in the second model incorporating only sociodemographic controls, the odds of the digitally confident reporting COVID-19 comprehension are 1.88 times that of the digitally underconfident (*p* < .001). In Model 3, which adjusts for sociodemographic controls and general anxiety, the variable digital confidence continues to have a statistically significant relation with COVID-19 comprehension (*OR* = 1.91, *p* < .001).

In the full model (Model 4), this association holds despite the partial confounding of the explanatory variable by education. The statistically significant relationship between digital confidence and COVID-19 comprehension persists in Model 4, even though both income and education are entered into the model (*OR* = 1.731, *p* < .01, *p* < .01). Expressed in terms of AMEs, the digitally confident have a 6.7% higher probability of reporting COVID-19 comprehension, as contrasted with their digitally underconfident counterparts (AME = 0.067, *p* < .01, Model 4).

**Figure 2.**
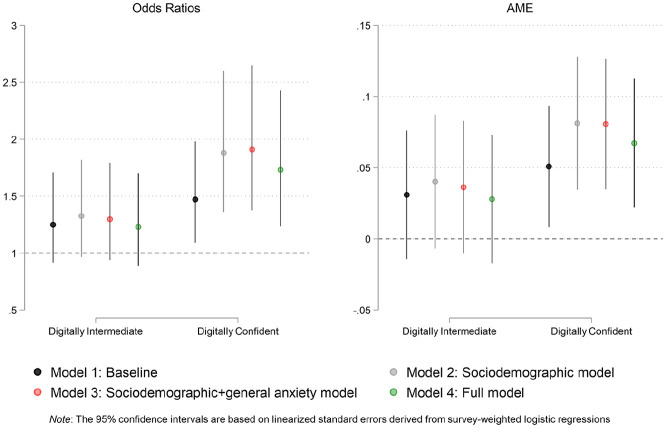
Coefficient plots for survey-weighted logistic models (COVID-19 comprehension).

Figure 2 shows the coefficients from the full model as given in odds ratios (left panel) as well as AMEs (right panel). Given these results, it is clear that the gap between the digitally confident and the digitally underconfident achieves statistical significance regardless of how the coefficients are calculated.

### Results for COVID-19 Comprehension: Controls in Full Model

In Tables 4 and 5, we present the odds ratio estimates and AMEs derived from the four survey-weighted logistic regression models. As before, the models are designed to predict the dichotomized outcome of COVID-19 comprehension as a function of the explanatory variable digital confidence and all control variables. With regard to the results interpreted in terms of odds ratios, we focus on the results from the full model. Here, we see that the sociodemographic factors of partner status, metropolitan status, and income do not exhibit a statistically significant relation with COVID-19 comprehension.

**Table 4.** Weighted Logistic Regression: COVID-19 Comprehension Dependent on Digital Confidence and Controls (Coefficients in Odds Ratios).

	(1)	(2)	(3)	(4)
	Baseline	Sociodemographic	Sociodemographic + anxiety	Full model
Digitally intermediate	1.249 (0.200)	1.326 (0.214)	1.297 (0.214)	1.229 (0.204)
Digitally confident	1.471* (0.223)	1.880*** (0.310)	1.909*** (0.319)	1.731** (0.299)
Age, years				
30-49		1.494** (0.219)	1.470** (0.218)	1.399* (0.210)
50-64		2.107*** (0.358)	1.971*** (0.337)	1.910*** (0.330)
65+		2.376*** (0.432)	2.040*** (0.376)	1.941*** (0.360)
Male		1.745*** (0.184)	1.556*** (0.167)	1.569*** (0.172)
Black non-Hispanic		0.494*** (0.077)	0.450*** (0.071)	0.468*** (0.074)
Hispanic		0.711* (0.106)	0.684* (0.101)	0.727* (0.108)
Race/ethnicity: Other		0.479*** (0.084)	0.464*** (0.084)	0.451*** (0.082)
Partnerless		0.987 (0.106)	1.054 (0.114)	1.070 (0.121)
Nonmetropolitan		1.308 (0.231)	1.343 (0.247)	1.409 (0.261)
Anxiety				
Most or all of the time (5-7 days)			0.331*** (0.055)	0.332*** (0.055)
Occasionally or a moderate amount of time (3-4 days)			0.492*** (0.071)	0.489*** (0.070)
Some or a little of the time (1-2 days)			0.866 (0.121)	0.862 (0.121)
Income				
$30-$74,999				0.993 (0.137)
$75,000+				1.030 (0.157)
Education				
2 Years college				1.036 (0.135)
4 Years college				1.415** (0.183)
Observations	9,404	9,404	9,404	9,404
*F*	3.590	11.748	15.031	11.738
*p*	.028	.000	.000	.000
Survey-F (Archer–Lemeshow Test)	0.000	1.246	0.453	0.719
Survey-P (Archer–Lemeshow Test)	1.000	0.261	0.906	0.692

*Note*. Exponentiated coefficients. Linearized standard errors derived from survey-weighted logistic regressions (in parentheses).

* *p* < .05. ^**^p < .01. ^***^p < .001.

**Table 5.** Weighted Logistic Regression: COVID-19 Comprehension Dependent on Digital Confidence and Controls (Average Marginal Effects).

	(1)	(2)	(3)	(4)
	Baseline	Sociodemographic	Sociodemographic + anxiety	Full model
Digitally intermediate	0.031 (0.023)	0.040 (0.024)	0.036 (0.024)	0.028 (0.023)
Digitally confident	0.051* (0.022)	0.081*** (0.024)	0.081*** (0.023)	0.067** (0.023)
Age, years				
30-49		0.057** (0.022)	0.052* (0.021)	0.045* (0.021)
50-64		0.096*** (0.023)	0.084*** (0.023)	0.079*** (0.022)
65+		0.107*** (0.024)	0.087*** (0.024)	0.080*** (0.023)
Male		0.066*** (0.012)	0.051*** (0.012)	0.052*** (0.012)
Black non-Hispanic		−0.092*** (0.024)	−0.103*** (0.024)	−0.098*** (0.024)
Hispanic		−0.039* (0.018)	−0.043* (0.018)	−0.035* (0.018)
Race/ethnicity: Other		−0.097*** (0.027)	−0.098*** (0.028)	−0.103*** (0.028)
Partnerless		−0.002 (0.013)	0.006 (0.012)	0.008 (0.013)
Nonmetropolitan		0.030 (0.018)	0.032 (0.018)	0.037* (0.018)
Anxiety				
Most or all of the time (5-7 days)			−0.146*** (0.024)	−0.144*** (0.024)
Occasionally or a moderate amount of time (3-4 days)			−0.082*** (0.017)	−0.083*** (0.017)
Some or a little of the time (1-2 days)			−0.014 (0.013)	−0.014 (0.013)
Income				
$30-$74,999				−0.001 (0.016)
$75,000+				0.003 (0.018)
Education				
2 Years college				0.004 (0.016)
4 Years college				0.039** (0.015)
Observations	9,404	9,404	9,404	9,404
*F*	3.590	11.748	15.031	11.738
*p*	.028	.000	.000	.000
Survey-F (Archer–Lemeshow Test)	0.000	1.246	0.453	0.719
Survey-P (Archer–Lemeshow Test)	1.000	0.261	0.906	0.692

*Note*. Standard errors derived from survey-weighted logistic regressions (in parentheses).

* *p* < .05. ^**^p < .01. ^***^p < .001.

Turning to the full model, as with the outcome of pandemic anxiety, age, race/ethnicity, gender, general anxiety, and education have a strong and statistically significant association with the outcome of COVID-19 comprehension. As reported in Model 4, respondents in the three older age brackets are more likely to report COVID-19 comprehension than respondents in the baseline age range of 18 to 29 years with the odds of comprehension rising as a function of age. For example, respondents 65+ are 1.94 times more likely to comprehend COVID-19 (*p* < .001). Gender also exhibits statistically significant effects on COVID-19 comprehension across all three models. In the full model, for example, the odds of reporting COVID-19 comprehension for male respondents are significantly higher than those for female respondents (*OR* = 1.57, *p* < .001, Model 4). Where general anxiety is concerned, the greater the frequency of nonpandemic anxiety, the lower the odds of COVID-19 comprehension. In Model 4, members of the two highest frequency general anxiety categories have lower odds of COVID-19 comprehension. For example, those experiencing anxiety 3 to 4 days per week are .489 times less likely (*p* < .001) to report COVID-19 comprehension than those in the baseline general anxiety frequency category.

By contrast to the first outcome of pandemic anxiety, race/ethnicity are a significant predictor of the second outcome of COVID-19 comprehension. When compared with the baseline category White non-Hispanic, respondents who are Black non-Hispanic (*OR* = 0.468, *p* < .001, Model 4), Hispanic (*OR* = 0.727, *p* < .05, Model 4), and Race/Ethnicity Other (*OR* = 0.451, *p* < .001, Model 4) all are less likely to report COVID-19 comprehension. Finally, also diverging from pandemic anxiety, those in the highest educational group (4-year college or postgraduate) are more likely to report COVID-19 comprehension compared with the baseline group comprising respondents with a high school education or less (*OR* = 1.42, *p* < .01, Model 4). The result for the outcome of COVID-19 comprehension therefore diverges from the models predicting pandemic anxiety. Where COVID-19 comprehension is concerned, both race/ethnicity and having at least a 4-year college degree achieve statistical significance as predictors.

### Robustness and Sensitivity Analyses

Several primary robustness and sensitivity analyses were conducted to address potential concerns with the models. These analyses were performed on a separate analytic data set generated by removing the variable with the largest number of missing values. Since missing values on income (*n* = 351) accounted for roughly half of the missing values on all analytic variables (*n* = 735), we utilized the data set without the income variable (*n* = 9,755) to perform these diagnostic analyses.

Using this data set, postestimation diagnostic checks on the full model were performed for each of the outcome variables. An examination of residual diagnostic plots was carried out to confirm the absence of unduly influential observations, as well as the absence of systematically trending residuals. We also computed standard logistic models using the maximum likelihood estimator and carried out the Hosmer–Lemeshow Goodness-of-Fit Test on the unweighted data. These procedures did not generate any evidence undermining the claim of a sufficiently good fit between the models and the data. We also calculated the variance inflation factor for the predictors and found no problematic multicollinearity among the predictors.

Using the complete analytic data set, linear probability models were also calculated on the basis of the full model specifications, and these models agreed sufficiently with the logistic models. In addition, to guard against specification error where the outcome variable of pandemic anxiety was concerned, we ran the models using the raw outcome variable given as a four-level categorical variable. Using this categorical outcome variable, we used a weighted ordinal logistic regression to check for consistency with the binary logistic models. This procedure revealed sufficient consistency across the two types of models to warrant the use of the simpler logistic regression with the dichotomous outcome. Finally, to check whether the specification of the categorical predictors with more than four levels had an impact on the findings, we also ran expanded models with the raw predictors. These expanded models included the fine-grained version of the two categorical independent variables partner status and education. This procedure allowed us to check for inconsistencies attributable to the specification of these two predictors. The results from these expanded models were consistent with those yielded by the compact models presented in the findings section.

## Summary of Findings

In this study, we show that digital confidence has important implications for two key effects of the pandemic: anxiety and information comprehension. The findings reveal that digitally confident individuals exhibit lower probability of suffering from pandemic anxiety and higher odds of comprehending information on the COVID-19 crisis. These findings hold true across all model specifications with sociodemographic, socioeconomic, and/or general anxiety controls. While the control variables can be considered partial confounders, their confounding effects are very small, and there is a substantial remaining effect due to digital confidence. With the exception of age, the impact of digital confidence is largely robust to the introduction of sociodemographic and socioeconomic status control variables in the models, as well as general anxiety. The logistic models therefore supply evidence supporting both of the hypotheses set out in the beginning of the article. Regarding Hypothesis 1, the digitally confident exhibit lower probability of experiencing physical symptoms of anxiety related to COVID 19, relative to respondents who belong to the digitally underconfident comparison group. Concerning Hypothesis 2, the digitally confident exhibit higher probability of “having a handle” on the COVID-19 crisis relative to the digitally underconfident.

## Limitations

It should be noted that the estimates generated by the models may reflect the timing of the data collection. Since the ATP Wave 66 data were collected in April 2020 during the first wave of the COVID-19 pandemic, we are not able to investigate whether the observed associations persisted into the second wave of the pandemic when the crisis worsened in the United States. Nor does the data set allow us to measure the long-term effects of digital confidence on pandemic anxiety and COVID-19 comprehension in terms of lasting effects. Both of these limitations can hopefully be addressed with future waves of the ATP survey by Pew should they incorporate the same questions about pandemic anxiety, COVID-19 comprehension, and digital confidence.

## Contributions

We have taken up the challenge of uncovering associations tying together responses to the COVID-19 crisis—specifically pandemic anxiety and COVID-19 comprehension—and digital inequality. Based on the analysis, digital inequality in the form of underconfidence emerges as an important intensifier of anxiety and incomprehension, key facets of individuals’ responses to the ongoing crisis. It would therefore be a mistake to neglect this aspect of inequality in future studies of the COVID-19 crisis and digital inequalities writ large.

As we have shown, the probability of suffering from pandemic anxiety and COVID-19 comprehension depend on the extent to which individuals use the internet more frequently and have acquired digital confidence. One of these two aspects of digital advantage, in other words, is insufficient to make a difference to these two outcomes. Thus, we can say that it is the co-occurrence of these two aspects of digital advantage which proves decisive for explaining differences in pandemic anxiety and COVID-19 comprehension.

Formulating this point in the terminology of the digital inequality literature, the more anxious and less COVID-19 comprehending individuals are those likeliest to lack access or exposure to digital resources and the digital confidence to use them effectively. Mere access to digital resources does not ameliorate either pandemic anxiety or COVID-19 comprehension, as membership in the intermediate category of digital “dabblers” generates no statistically significant gain when it comes to reducing pandemic anxiety or increasing COVID-19 comprehension.

Why would membership in the digitally confident group make such a difference? Thanks to their comfort and ability to use the internet frequently, the digitally confident are far better positioned to use digital resources to mitigate the effects of the COVID-19 pandemic studied here. With regards to COVID-19 comprehension, the digitally confident are better able to surf the internet at will, find and extract important information on the COVID-19 crisis, and deploy it effectively. In terms of pandemic anxiety, the digitally confident experience greater digital agency in their ability to use informational resources to grapple with the virus, shield themselves, and mitigate risk. Experiencing greater agency, the digitally confident are less exposed to anxiety-inducing feelings produced by loss of control.

By contrast, the digitally underconfident are in a poorer position to find, extract, and deploy information related to the crisis. Awash in an overwhelming sea of information, but without digital skills and resources to manage it effectively, the digitally underconfident flounder in an unmanageable flood of information. Without the access or digital confidence to decide what information valuable and/or reliable, they struggle to achieve comprehension. This struggle may lead to a loss of agency and self-efficacy, especially given the potential life and death consequences of COVID-19. The loss of agency understandably intensifies the classic physical symptoms of anxiety: sweating, trouble breathing, nausea, or a pounding heart. Indeed, as we know from previous studies, individuals lacking confidence in their ability to find relevant information online and determine its veracity are more predisposed to stress even in less turbulent times (Huang et al., 2015; Robinson, 2018; van Dijk, 2005). From this angle of view, the COVID connection between digital confidence, comprehension of information about the pandemic, and somatic symptoms of anxiety becomes not only evident but inescapable.

## Implications

Given that the analysis supports both hypotheses showing the statistically significant impact of digital confidence on both outcomes: pandemic anxiety and COVID-19 comprehension, the study builds an important bridge between the established literature on the digital divide literature and the nascent body of research on the COVID-19 pandemic.

Our research is the first to establish a link between somatized stress and digital inequality. It shows that digital underconfidence can not only lead to a problematic lack of comprehension concerning the crisis, but can also affect bodily well-being itself by aggravating somatic symptoms of anxiety. From a broader perspective, the findings that digital confidence makes a difference to the two outcomes, even taking these other measures of vulnerability into account. Therefore, a number of fields would benefit by further exploring digital confidence as a key aspect of vulnerability.

Our findings point to critical ways in which the digitally disengaged are disadvantaged both directly and indirectly during this ongoing crisis. Not only are the digitally underconfident directly disadvantaged because they face challenges in availing themselves of opportunities to work, communicate, and consume through online channels—but they also suffer unequally from ill effects surfacing when they struggle to weather the waves of (mis)information inundating the public. For this reason, future studies cannot omit digital confidence if they wish to fully account for individuals’ responses to crises and disasters.

During a time when the U.S. and the world are facing a cascading and long-lasting crisis with deleterious consequences for the emotional health of the public, the lack of digital confidence emerges as yet another unequally distributed dimension of vulnerability. Therefore, it is not enough to ameliorate the longstanding sociodemographic, economic, and psychological vulnerabilities of the most impacted segments of the population. Nor is sufficient to close existing digital divides simply in terms of access and skills. We must also work on building digital confidence as critical, and understudied, facet of social resilience.

Going forward, future policy must address both dimensions of digital confidence. It is not enough to provide mere access to digital networks and devices. Policy must also ensure adequate literacy and training opportunities so that all members of society may acquire confidence in their ability to use digital tools effectively for the pandemic and future crises. Future work must probe these issues further to equip policy makers with actionable findings to mitigate the suffering of vulnerable populations who are disproportionately harmed by the pandemic. From this perspective, digital confidence should be a capability that is nurtured by a robust policy agenda and infrastructure. When this is achieved, digital confidence will equip all of us to better cope with the crises of the future.

## Notes

1 For additional information on the data set, see the Pew description of their recruitment methodology, consenting process, details on variable construction, and instructions for design weights provided on their web pages including: • https://www.pewresearch.org/methods/u-s-survey-research/questionnaire-design/ • https://www.pewresearch.org/politics/2020/09/10/voters-attitudes-race-gender-methodology • https://www.pewresearch.org/pathways-2020/sourceuse_19/digital_savviness/us_adults • https://www.journalism.org/2020/01/24/election-news-pathways-project-frequently-asked-questions/#measuring-digital-savviness

2 We compared descriptive statistics for the weighted and unweighted variables the unweighted and weighted distributions are very similar for the majority of the variable categories. Those few exceptions in which the survey weights compensate for a smaller turnout include an adjustment for those in two response categories under age and income, respectively: (1) 18 to 29 years and (2) less than $30,000 annual income.

3 Although we do not read the literature to indicate that our final models have omitted any variables with strong biasing impacts, we acknowledge an ongoing discussion in the field Allison, 1999). Following Mood’s recommendation that: “it is often advisable to report results using more than one type of estimate” (Mood, 2009: 80), we calculate both odds ratios and average marginal effects for each coefficient in our models.
